# Morin Attenuates Diclofenac‐Induced Hepatocellular Death Injury via Nrf2/Ho‐1/NQO1, Beclin‐1/LC3A/LC3B and p53/Bax/Caspase Signalling Pathways

**DOI:** 10.1002/jbt.70372

**Published:** 2025-06-23

**Authors:** Nurhan Akaras, Hasan Şimşek, Cihan Gür, Sefa Küçükler, Mustafa İleritürk, Fatih Mehmet Kandemir

**Affiliations:** ^1^ Department of Histology and Embryology Faculty of Medicine Aksaray University Aksaray Turkey; ^2^ Department of Physiology Faculty of Medicine Aksaray University Aksaray Turkey; ^3^ Department of Medical Laboratory Techniques Vocational School of Health Services Atatürk University Erzurum Turkey; ^4^ Department of Veterinary Biochemistry Faculty of Veterinary Atatürk University Erzurum Turkey; ^5^ Department of Animal Science Horasan Vocational College Atatürk University Erzurum Turkey; ^6^ Department of Medical Biochemistry Faculty of Medicine Aksaray University Aksaray Turkey

**Keywords:** apoptosis, autophagy, diclofenac, liver, morin, oxidative stress

## Abstract

Diclofenac (DF), a nonsteroidal and anti‐inflammatory drug, has limited use due to its adverse effects on the liver. On the other hand, morin, a bioflavonoid, has biological and pharmacological properties. This study aims to investigate whether morin may protect against diclofenac‐induced liver toxicity. For this purpose, morin (50 or 100 mg/kg) treatment was given orally to the rats for 5 days, and DF (50 mg/kg) was administered intraperitoneally on the 4th and 5th days of the study. Molecular, biochemical, immunohistochemical and histological methods were used to investigate cyclooxygenase enzymes, oxidative stress, apoptosis and autophagy in liver tissue. According to the data obtained, it was observed that DF caused oxidative stress, autophagy and apoptosis damage in liver tissues. Morin showed antioxidant properties, causing a decrease in MDA in hepatic tissue, an increase in the activities of endogenous antioxidants (glutathione peroxidase, superoxide dismutase and catalase) and GSH, HO‐1, Nrf2 and NQO1 mRNA levels. Moreover, morin reversed the changes in the levels of apoptotic and autophagic parameters such as bax, bcl‐2, cytochrome c, p53, Apaf‐1, caspase‐3, caspase‐6, caspase‐9, beclin‐1, LC3A, LC3B, MAPK14, MAP15, JNK. When the histopathological analysis results were examined, degenerative changes occurred in the livers of rats administered DF, while morin administration showed a morphological structure close to the control group. As a result, it was determined that oxidative stress, autophagy and apoptosis caused by DF were suppressed by morin, thus protecting the liver tissue from damage.

## Graphical abstract

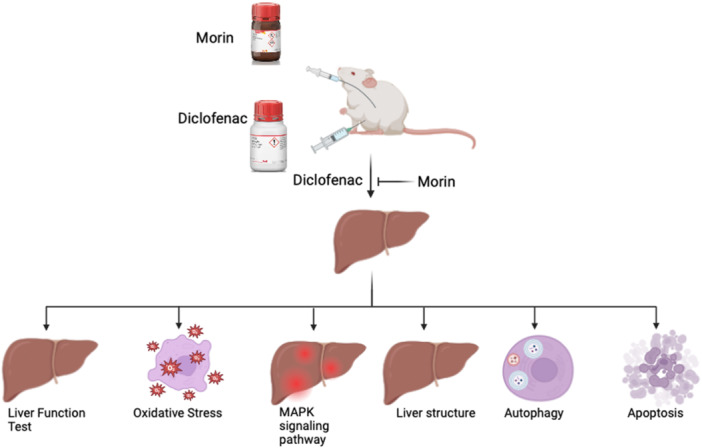

## 1 Introduction

Diclofenac (DF) is a phenylacetic acid‐derived nonsteroidal anti‐inflammatory drug (NSAID) that is the most widely used and available over‐the‐counter worldwide for its anti‐inflammatory, antipyretic and analgesic properties [1]. After DF is taken into the body, it is absorbed from the intestines and reaches its highest concentration after approximately 2 h. This drug, metabolized in the liver, is converted into hydroxydiclofenac, then conjugated with sulfate and glucuronic acid and excreted through the kidney. This makes the liver an important target organ in drug‐induced injury (DILI), since the liver is involved in the defoxification and excretion of drugs and metabolites [1, 2, 3, 4]. Although DF is a well‐tolerated drug, it is well known to cause DILI [5]. DILI is the main cause of liver dysfunction, liver injury, liver failure and associated mortality [6]. Although 6 to 8 out of 100,000 cases of DF‐induced hepatic damage are reported each year, this number is actually estimated to be 20 times higher. DF has been reported to be the third drug with the highest risk of side effects compared with other nonsteroidal anti‐inflammatory drugs [7]. Inhibition of Cyclooxygenase‐2 (COX‐2) by suppressing prostaglandin synthesis from arachidonic acid is a known mechanism of action of DF [8]. Although the underlying mechanisms of DF‐induced DILI are unknown, it is an adverse reaction caused by an increase in cellular oxidative stress as a result of drug biotransformation in the liver [9]. Recent evidence suggests that inhibition of COX enzymes also induces oxidative stress [9, 10]. It is among the pathways that are largely involved in mitochondrial damage and immune‐mediated damage, along with the molecular mechanism mediated by oxidative stress. Considering these underlying mechanisms, natural agents with antioxidant properties in liver toxicity have become a subject of interest [5].

Flavonoids are phenolic compounds that are naturally found in many vegetables and fruits and have important effects on health. Previous studies have shown that flavonoids can be used as protective agents against hepatotoxicity due to their anti‐inflammatory, antiapoptotic and anti‐autophagic properties [5, 6, 8, 11]. Morin (3,5,7,2′,4′‐pentahydroxyflavone) is a member of the *Moraceae* family, abundant in white berries, cranberry twigs and Chinese herbs [12, 13, 14]. Many in‐vitro and in‐vivo studies have revealed that morin has antioxidant, anti‐inflammatory, antidiabetic, anticarcinogenic, antiapoptotic and antiproliferative effects. Morin is not only a free radical scavenger but also an inhibitor of enzymes that catalyze free radical production [13, 15, 16]. Since these conditions create an important chance for morin and anti‐inflammatory drugs to be taken together, it is important to study the interaction between morin and NSAID's.

Although morin is known to be antiapoptotic and anti‐inflammatory, the molecular mechanisms underlying this response are still unclear. The aim of this study was to investigate the antioxidant, anti‐inflammatory and antiapoptotic properties of morin against DF‐induced toxicity in rat liver and the protective effects of morin on tissue damage biochemically, immunohistochemically and histopathologically.

## 2 Materials and Methods

### 2.1 Chemicals and Animals

Diclofenac was purchased from Abdi Ibrahim (75 mg/3 mL). Morin (302.24 g/mol; Cas no: 654055‐01‐3) and all chemicals were purchased from Sigma (Aldrich (St Louis, MO) in high analytical purity. In this study, 35 male Wistar albino rats aged 10–12 weeks and weighing 220–250 g were used as experimental animals. All animal experiments were obtained from KONÜDAM Experimental Medicine Application and Research Centre (Konya/TURKEY). Rats were housed in standard cages with a temperature of 23°C−25°C, 55% humidity, 12 h light/12 h dark cycle and fed ad libitum with standard rat chow and tap water. The study was approved by Necmettin Erbakan University Animal Experiments Local Ethics Committee (no.2022‐056). Before starting the experiment, all animals were randomly divided into five groups with seven rats in each group. DF and Morin dosages were used in accordance with previous studies [17, 18].

1. Control Group: saline was administered orally daily for 5 days.
2. Morin Group: 100 mg/kg orally once daily for 5 days.
3. Diclofenac Group (DF): Diclo 50 mg/kg was administered intraperitoneally (i.p.) on Days 4 and 5.
4. Diclofenac + Morin 50 mg/kg (DF+Morin 50): Diclo 50 mg/kg was administered i.p. on Days 4 and 5. Morin was administered 50 mg/kg orally daily for 5 days.
5. Diclofenac + Morin 100 mg/kg (DF+Morin 100): Diclo 50 mg/kg was administered i.p. on Days 4 and 5. Morin was administered 100 mg/kg orally once daily for 5 days.

Animal blood and rat livers were collected 24 h after the last drug administration under light sevoflurane anaesthesia. Blood and liver tissues were used for histological, biochemical and molecular analysis. Histopathological and immunohistochemical analyses were performed in Aksaray University Histology laboratory, biochemical and molecular analyses were performed in Aksaray University Faculty of Medicine Biochemistry laboratory and Atatürk University Faculty of Veterinary Medicine Biochemistry laboratory.

### 2.2 Measurements of Lipid Peroxidation and Antioxidants

To measure the degree of lipid peroxidation, malondialdehyde (MDA) level was measured by the method developed by Placer et al. and concentrations were expressed as nmol/g tissue [19]. To evaluate the antioxidant status, Glutathione (GSH) levels were measured by Sedlak and Lindsay method and expressed as nmol/g tissue [20]. Glutathione peroxidase (GPx) activity was measured according to the method developed by Lawrence and Burk and expressed as U/g protein [21]. Superoxide dismutase (SOD) activity measurements of antioxidant enzymes were determined according to the method of Sun et al. and given in U/g protein [22]. Catalase (CAT) activity was determined by the Aebi method measuring the rate of degradation of hydrogen peroxide per unit time and expressed as catal/g protein [23]. To calculate enzyme activities, total protein was determined according to the method of Lowry et al. [24].

### 2.3 Total RNA Isolation and cDNA Synthesis

Total RNAs were isolated from liver tissues using QIAzol Lysis Reagent (79306; Qiagen). The concentrations of RNAs were determined on a NanoDrop (BioTek Epoch) device. RNAs were then converted into cDNAs using the iScript cDNA Synthesis Kit (Bio‐Rad).

### 2.4 RT‐PCR Analyzes

The cDNAs previously synthesised from RNAs in the RT‐PCR step included Bcl‐2, Apoptotic protease activating factor‐1 (Apaf‐1), Caspase‐3, Bax, P53, Caspase‐6, Caspase‐9, nuclear factor erythroid 2 like 2 (Nrf2), heme oxygenase 1 (HO‐1), NAD(P)H: quinone oxidoreductase 1 (NQO1), light chain 3 A (LC3A), light chain 3B (LC3B), Beclin‐1, COX‐2, mitogen‐activated protein kinase 14 (MAPK14), mitogen‐activated protein kinase 15 (MAPK15), C‐Jun N‐terminal kinase (JNK) and β‐Actin genes were reacted with iTaq Universal SYBR Green Supermix (BIORAD) in the presence of reverse and forward primers (Table 1) in Rotor‐Gene Q (Qiagen). The volumes and temperature cycles used for the reaction were prepared according to the manufacturer's instructions. After completion of the cycles, genes were normalised to Beta‐Actin by the 2^‐ΔΔCT^ method [25].

**Table 1:** Primer sequences.

Gene	Sequences (5′‐3′)	Length (bp)	Accession No
*Bcl‐2*	F: GACTTTGCAGAGATGTCCAG R: TCAGGTACTCAGTCATCCAC	214	NM_016993.2
*Apaf‐1*	F: ACCTGAGGTGTCAGGACC R: CCGTCGAGCATGAGCCAA	192	NM_023979.2
*Caspase‐3*	F: ACTGGAATGTCAGCTCGCAA R: GCAGTAGTCGCCTCTGAAGA	270	NM_012922.2
*Bax*	F: TTTCATCCAGGATCGAGCAG R: AATCATCCTCTGCAGCTCCA	154	NM_017059.2
*P53*	F: GCGCTTCGAGATGTTCCGA R: AGACTGGCCCTTCTTGGTCT	121	NM_030989.3
*Caspase‐6*	F: GAACGAACGGACCTGTGGA R: CAGTCCAGCTCTGTACCTCG	124	NM_012922.2
*Caspase‐9*	F: ACGTGAACTTCTGCCCTTCC R: GGTCGTTCTTCACCTCCACC	117	NM_031632.2
*Nrf2*	F: TTTGTAGATGACCATGAGTCGC R: TCCTGCCAAACTTGCTCCAT	161	NM_031789.2
*HO‐1*	F: ATGTCCCAGGATTTGTCCGA R: ATGGTACAAGGAGGCCATCA	144	NM_012580.2
*NQO1*	F: CTGGCCAATTCAGAGTGGCA R: GATCTGGTTGTCGGCTGGAA	304	NM_017000.3
*LC3A*	F: GACCATGTTAACATGAGCGA R: CCTGTTCATAGATGTCAGCG	139	NM_199500.2
*LC3B*	F: GAGCTTCGAACAAAGAGTGG R: CGCTCATATTCACGTGATCA	152	NM_022867.2
*Beclin‐1*	F: TCTCGTCAAGGCGTCACTTC R: CCATTCTTTAGGCCCCGACG	198	NM_053739.2
*COX‐2*	F: AGGTTCTTCTGAGGAGAGAG R: CTCCACCGATGACCTGATAT	240	NM_017232.3
*MAPK14*	F: GTGGCAGTGAAGAAGCTGTC R: GTCACCAGGTACACATCGTT	170	NM_031020.2
*MAPK15*	F: TGTTTGAGTCCATGGACACC R: GCATCCAATAGAACGTTGGC	169	NM_173331.2
*JNK*	F: GAATCAGACCCATGCTAAGC R: CCATGAGCTCCATGACTATG	149	NM_053829.2
*β‐Actin*	F: CAGCCTTCCTTCTTGGGTATG R: AGCTCAGTAACAGTCCGCCT	360	NM_031144.3

### 2.5 Immunohistochemical Analysis

3 µm thick sections taken from liver tissues were prepared for staining after being passed through xylene and alcohol series. Antigen retrieval was performed by keeping the sections in ethylene diamine tetra acetic acid (EDTA) buffer. Then, it was kept in 3% hydrogen peroxide for 18 min. The sections, in which endogenous peroxidase activity was inhibited, were washed with phosphate‐buffered saline (PBS) and then treated with protein block for 10 min. The primary antibody diluted with PBS was dropped onto the sections and kept in the refrigerator (+ 4°C) overnight. Then, after washing with PBS three times for 5 min, they were treated with secondary antibody and Strepto Biotin, respectively. After each procedure, DAB solution was dropped on the sections washed with PBS and waited until a brown color appeared. The sections were treated with Harris Hematoxylin for 2 min and washed again with PBS. The tissues were kept in 96% alcohol for 5 min, in absolute alcohol twice for 5 min, and in xylene twice for 5 min and then covered with entellan. Antibodies used for immunohistochemical staining: caspase‐3 (sc56053) and 8‐OHdG (sc66036). Grading by immunohistochemical staining was based on a score of 0–3 for each section. All samples were scored as follows: 0: none, 1: minimal staining, 2: moderate staining, and 3: extensive staining. Seven sections were randomly selected from each group and the staining intensity was evaluated with Image J software (Image J, version 1.46a, NIH, Bethesda, MD, USA).

### 2.6 Histopathological Analysis

Liver tissues were kept in 10% formalin solution for fixative purposes for 24 h. Fixed tissues were first passed through increasing grades of alcohol (70%–100%) and then cleared in xylene. As the last step in the tissue tracking phase, 5 µm thick sections were taken from the prepared paraffin blocks using a microtome. The prepared sections were stained with Hematoxylin‐Eosin (H&E) for general histological evaluation. Stained sections obtained from liver tissues were examined under a light microscope (Olympus Cx43; Japan) and photographed. Random areas were selected for each animal and tissue damage was assessed using a blinded method. The evaluation of differences between the groups in liver sections was based on the evaluation of the severity of pathological changes, including factors such as necrosis, hyperemia and hemorrhage in interstitial vessels, and inflammatory cell infiltration. A grading system was used that assigned points according to the severity of histopathological lesions: 0 none, 1 mild, 2 moderate, and 3 indicated severe.

### 2.7 Statistical Analysis

Statistical analysis of biochemical findings was performed by one‐way ANOVA and Tukey HSD test was used to determine the relationship between the groups. Results are presented as Mean ± Standard Deviation of Mean. The results of histological examination were analysed by nonparametric Kruskal‐Wallis test and Mann‐Whitney U test for comparison of paired groups. Statistical significance level was accepted as *p* < 0.05.

## 3 Results

### 3.1 Effect of Morin and DF on Serum Liver Biomarkers

Aspartate aminotransferase (AST), alanine transaminase (ALT) and alkaline phosphatase (ALP) blood results of DF and morin treated rats are presented in Table 2. AST, ALT and ALP serum levels in DF‐treated rats were significantly increased compared to the control group. Morin treatment at 50 and 100 mg/kg significantly decreased these enzyme levels compared with the DF group (*p* < 0.05).

**Table 2:** Oxidative stress markers in liver tissue and ALT, AST and ALP levels in serum.

Paremeters	Control	Morin	DF	DF + Morin 50	DF + Morin 100
MDA (nmol/g tissue)	35.08 ± 3.77	35.45 ± 3.31^###^	82.57 ± 4.52***	64.99 ± 3.89***^/###/⁎⁎^	56.63 ± 3.29***^/###^
GSH (nmol/g tissue)	8.61 ± 0.37	8.70 ± 0.40^###^	3.41 ± 0.25***	4.77 ± 0.31***^/###/⁎⁎⁎^	7.05 ± 0.34***^/###^
SOD (U/g protein)	28.89 ± 2.85	28.95 ± 2.74^###^	13.04 ± 1.86***	18.54 ± 2.06***^/##^	21.85 ± 2.50***^/###^
GPx (U/g protein)	39.92 ± 3.58	40.85 ± 3.21^###^	17.26 ± 1.99***	25.58 ± 2.14***^/###/⁎⁎^	32.14 ± 3.00***^/###^
CAT (catal/g protein)	41.01 ± 3.57	42.31 ± 3.36^###^	21.31 ± 2.31***	27.38 ± 3.09***^/##/⁎⁎^	34.41 ± 2.70**^/###^
ALT (U/L)	36.71 ± 5.15	38.29 ± 5.09^###^	96.43 ± 11.32***	66.86 ± 9.77***^/###/⁎⁎^	48.14 ± 5.93^###^
AST (U/L)	94.57 ± 9.83	95.71 ± 10.50^###^	239.14 ± 15.48***	181.14 ± 18.47***^/###/⁎⁎⁎^	131.71 ± 13.47***^/###^
ALP (U/L)	76.00 ± 7.87	73.29 ± 7.99^###^	177.00 ± 15.73***	147.00 ± 11.42***^/###/⁎⁎^	118.57 ± 14.02***^/###^

*Note:* Control vs others: *p < 0.05, **p < 0.01, ***p< 0.001, DF vs others: #*p* < 0.05, ##*p*< 0.01, ###*p* < 0.001, DF + Morin 50 vs DF + Morin 100: ^⁎^ *p* < 0.05, ^⁎⁎^ *p* < 0.01, ^⁎⁎⁎^ *p* < 0.001.

### 3.2 Effect of Morin on DF‐Induced Oxidative Stress

Malondialdehyde levels and antioxidant enzyme activities of liver tissues are given in Table 2. When the MDA level of liver tissues was evaluated, it was determined that DF administration increased compared to the control group, which was accepted as the reference value. It was concluded that morin administration decreased MDA levels in a dose‐dependent manner (*p* < 0.05). SOD, CAT and GPx antioxidant enzyme activities and GSH levels were decreased in the DF group compared to the control group. However, in contrast to the DF group, these variables were statistically lower after Morin treatment (*p* < 0.001). In addition, antioxidant enzyme activities and GSH levels were significantly improved in the group given Morin 100 together with DF.

### 3.3 Effect of Morin on DF‐Induced Repressed Nrf‐2, HO‐1 and NQO1 Genes

**Figure 1:**
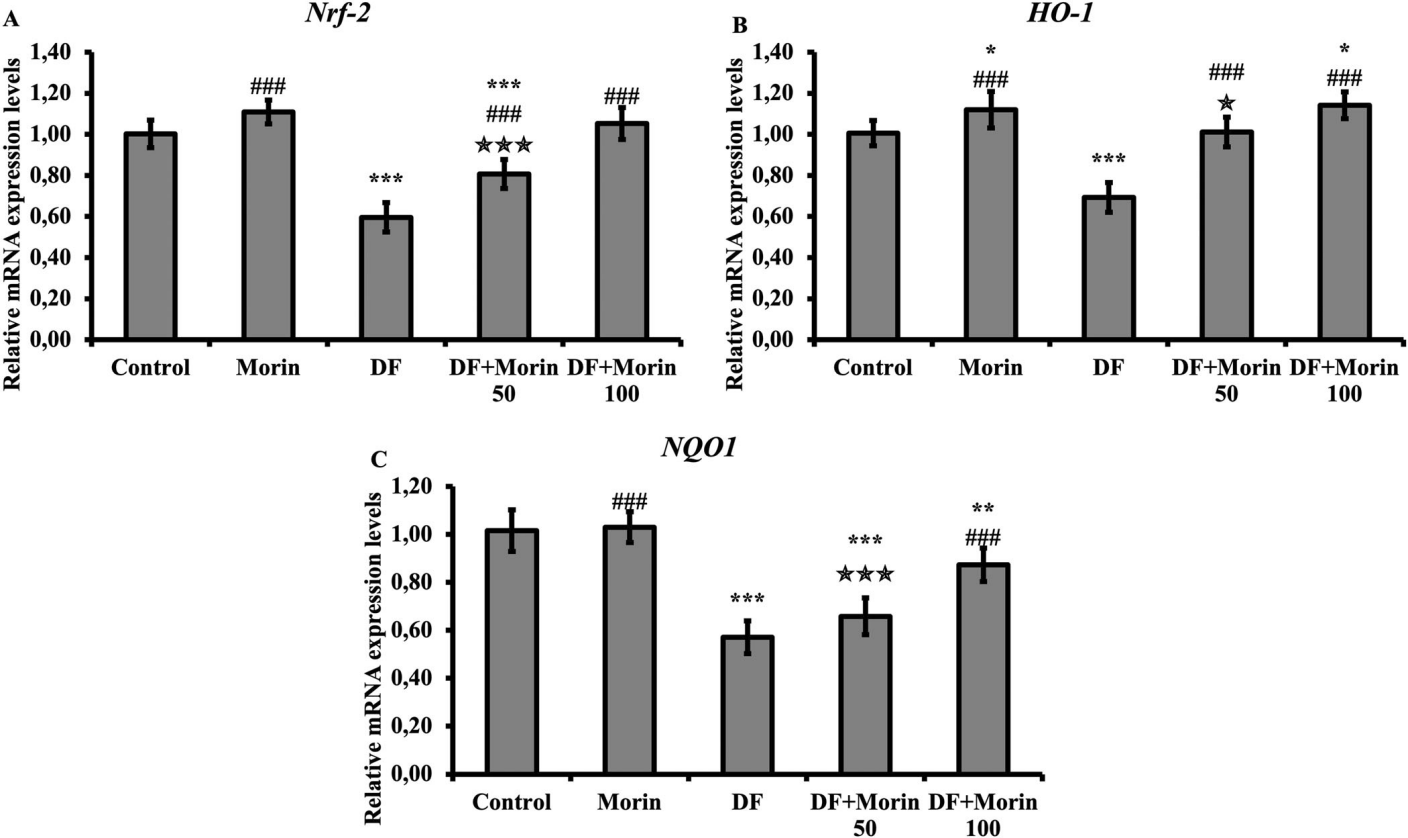
Effect of Morin and DF treatments on Nrf‐2, HO‐1, NQO1 mRNA expression levels in liver tissue. (A) Represent the relative mRNA expression levels of Nrf‐2, (B) Represent the relative mRNA expression levels of HO‐1, (C) Represent the relative mRNA expression levels of NQO1. Values are given as mean ± SD. Control vs others: *p < 0.05, **p < 0.01, ***p < 0.001, DF vs others: ^#^ *p* < 0.05, ^##^ *p* < 0.01, ^###^ *p* < 0.001, DF + Morin 50 vs DF + Morin 100: ^⁎^ *p* < 0.05, ^⁎⁎^ *p* < 0.01, ^⁎⁎⁎^ *p* < 0.001.

A significant decrease was observed in the levels of Nrf‐2, HO‐1 and NQO1 gene expressions in the DF group compared to the control group (*p* < 0.05). Treatment with both doses of MOR significantly increased the levels of these parameters compared with the DF alone group (*p* < 0.001, Figure 1A–C).

### 3.4 Effect of Morin on DF‐Induced Repression of COX‐2 Genes

**Figure 2:**
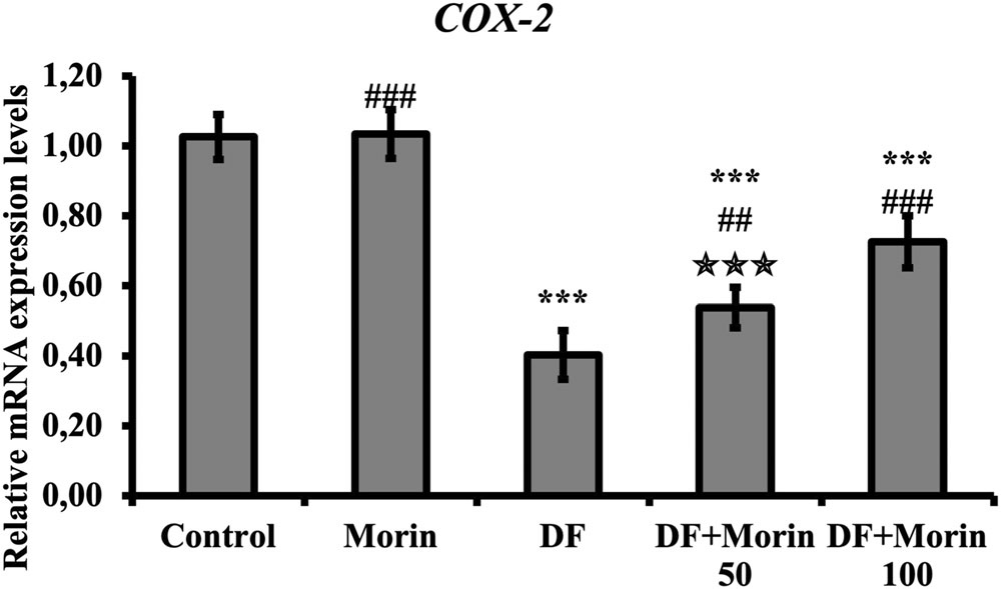
Protective effects of Morin and DF treatments on COX‐2 mRNA expression levels in liver tissue. Values are given as mean ± SD. Control vs others: *p < 0.05, **p < 0.01, ***p < 0.001, DF vs others: ^#^ *p* < 0.05, ^##^ *p* < 0.01, ^###^ *p* < 0.001, DF + Morin 50 vs DF + Morin 100: ^⁎^ *p* < 0.05, ^⁎⁎^ *p* < 0.01, ^⁎⁎⁎^ *p* < 0.001.

According to the data presented in Figure 2, a significant decrease in COX‐2 mRNA transcript levels, which is an important indicator of the inflammatory process, was observed in the DF group compared to the control group. COX‐2 expression was found to be increased in the DF + Morin 50 and DF + Morin 100 groups compared to the DF group (*p* < 0.001).

### 3.5 Effects of Morin on DF‐Induced Increased Beclin‐1, LC3A and LC3B Genes

**Figure 3:**
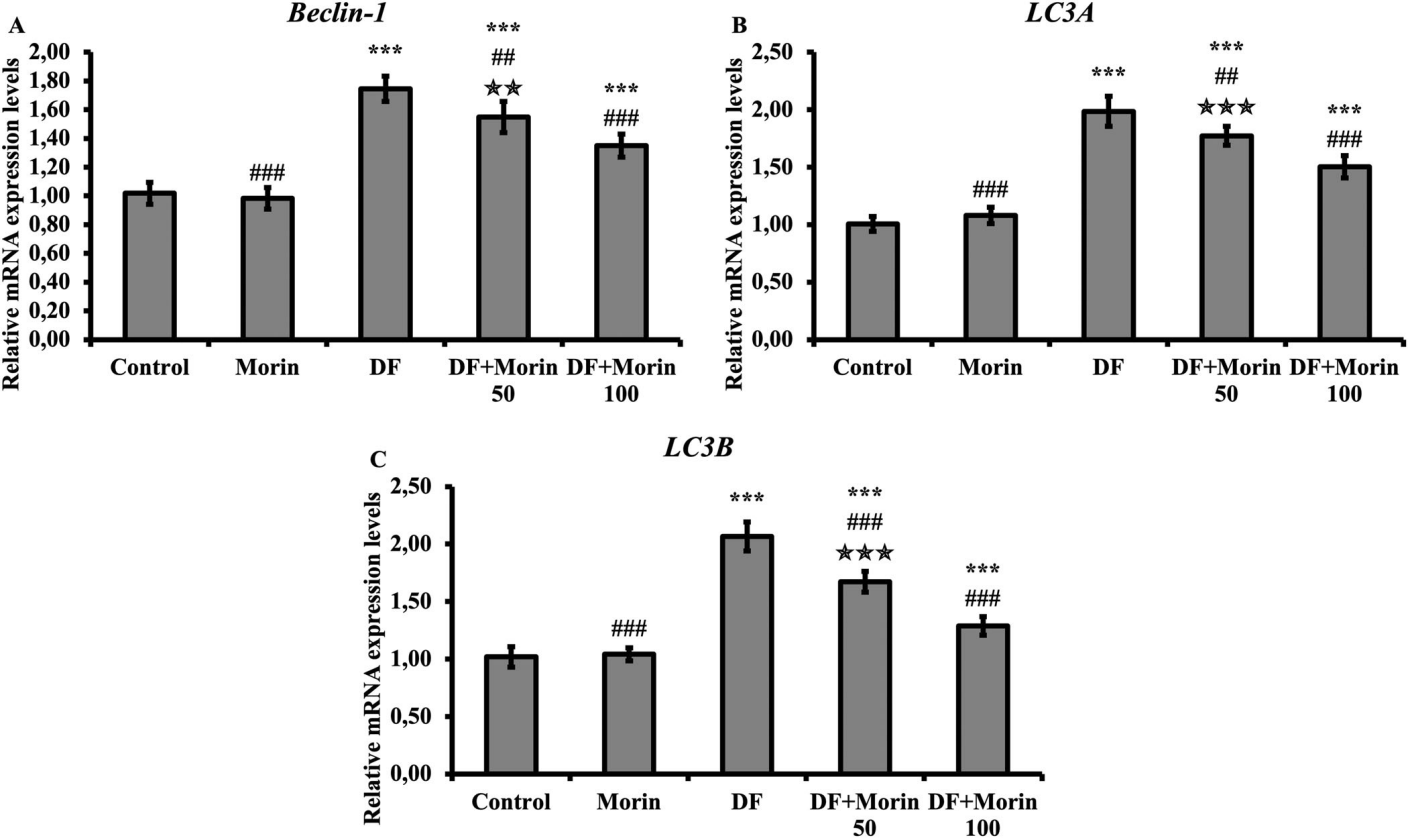
Protective effects of Morin and DF treatments on Beclin‐1, LC3A and LC3B mRNA expression levels in liver tissue. (A) Represent the relative mRNA expression levels of Beclin‐1, (B) Represent the relative mRNA expression levels of LC3A, (C) Represent the relative mRNA expression levels of LC3B. Values are given as mean ± SD. Control vs others: *p < 0.05, **p < 0.01, ***p < 0.001, DF vs others: ^#^ *p* < 0.05, ^##^ *p* < 0.01, ^###^ *p* < 0.001, DF + Morin 50 vs DF + Morin 100: ^⁎^ *p* < 0.05, ^⁎⁎^ *p* < 0.01, ^⁎⁎⁎^ *p* < 0.001.

In this study, the anti‐autophagic role of morin in DF toxicity was evaluated and presented in Figure 3. Although the levels of autophagic markers beclin‐1, LC3A and LC3B were significantly higher in the DF treated group compared to the control group, they were significantly decreased in the groups administered with 50 and 100 mg/kg morin.

### 3.6 Effects of Morin on DF‐Induced Increased Markers of Apoptosis

**Figure 4:**
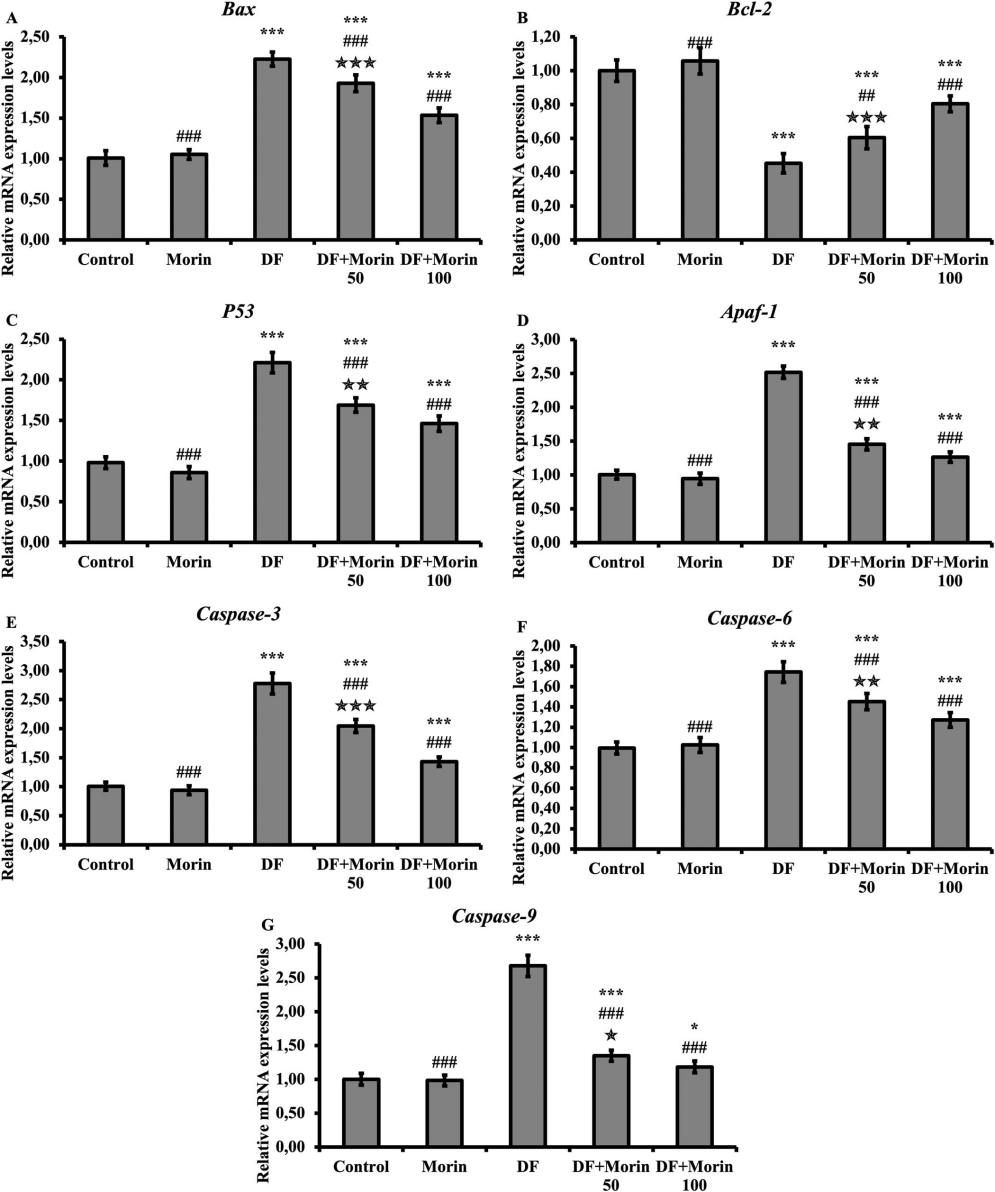
Protective effects of Morin and DF treatments on Bax, Bcl‐2, p53, Apaf‐1, caspase‐3, caspase‐6, caspase‐9 levels, mRNA expression levels in liver tissue. (A) Represent the levels of Bax, (B) Represent the levels of Bcl‐2, (C) Represent the levels of p53, (D) Represent the levels of Apaf‐1, (E) Represent the levels of caspase‐3, (F) Represent the levels of caspase‐6, (G) Represent the levels of caspase‐9. Values are given as mean ± SD. Control vs others: *p < 0.05, **p < 0.01, ***p < 0.001, DF vs others: ^#^ *p* < 0.05, ^##^ *p* < 0.01, ^###^ *p* < 0.001, DF + Morin 50 vs DF + Morin 100: ^⁎^ *p* < 0.05, ^⁎⁎^ *p* < 0.01, ^⁎⁎⁎^ *p* < 0.001.

**Figure 5:**
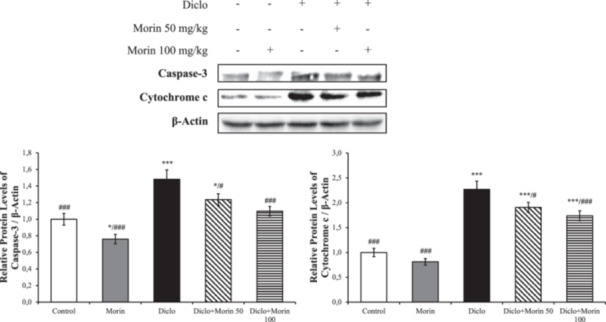
Protective effects of Morin and DF treatments on Caspase‐3 and Cytochrome c protein levels in liver tissue of rats. Values are given as mean ± SD. Control vs others: *p < 0.05, **p < 0.01, ***p < 0.001, DF vs others: ^#^ *p* < 0.05, ^##^ *p* < 0.01, ^###^ *p* < 0.001, DF + Morin 50 vs DF + Morin 100: ^⁎^ *p* < 0.05, ^⁎⁎^ *p* < 0.01, ^⁎⁎⁎^ *p* < 0.001.

The mRNA transcript levels of Bax, Bcl‐2, p53, caspase‐3, caspase 6, caspase 9 and Apaf‐1 in liver tissues are presented in Figure 4. According to the data, Bax, p53, caspase‐3, caspase 6, caspase 9 and Apaf‐1 expressions were upregulated and Bcl‐2 expression was downregulated as a result of DF administration. In DF + Morin 50 and DF + Morin 100 groups, Bax, p53, caspase‐3, caspase 6, caspase 9 and Apaf‐1 expression were suppressed and Bcl‐2 expression was induced. In addition, caspase‐3 and cytrocrome‐c levels were evaluated by western‐blot method and presented in Figure 5. According to the results, these protein levels increased in the DF‐treated group compared to the control, while caspase‐3 and cytrocrome‐c levels decreased with morin administration.

### 3.7 Effects of Morin on DF‐Induced Increased MAPK 14, MAPK 15 and JNK Genes

**Figure 6:**
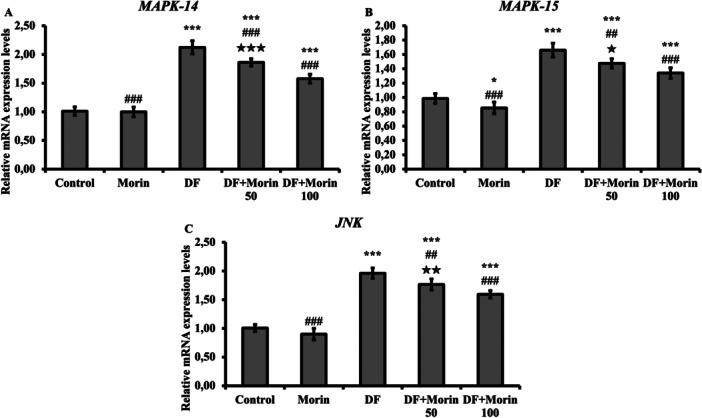
Protective effects of Morin and DF treatments on MAPK14, MAPK15 and JNK mRNA expression levels in liver tissue. (A) Represent the relative mRNA expression levels of MAPK14, (B) Represent the relative mRNA expression levels of MAPK15, (C) Represent the relative mRNA expression levels of JNK. Values are given as mean ± SD. Control vs others: *p < 0.05, **p < 0.01, ***p < 0.001, DF vs others: ^#^ *p* < 0.05, ^##^ *p* < 0.01, ^###^ *p* < 0.001, DF + Morin 50 vs DF + Morin 100: ^⁎^ *p* < 0.05, ^⁎⁎^ *p* < 0.01, ^⁎⁎⁎^ *p* < 0.001.

The effect of DF and Morin treatments on MAPK 14, MAPK 15 and JNK expression levels were analysed and presented in Figure 6. A significant increase in MAPK 14, MAPK 15 and JNK levels was observed in the DF treated group compared to the control group. The levels of MAPK 14, MAPK 15 and JNK markers were decreased in rats administered DF with morin compared to DF‐treated rats.

### 3.8 Immunohistochemical Results

**Figure 7:**
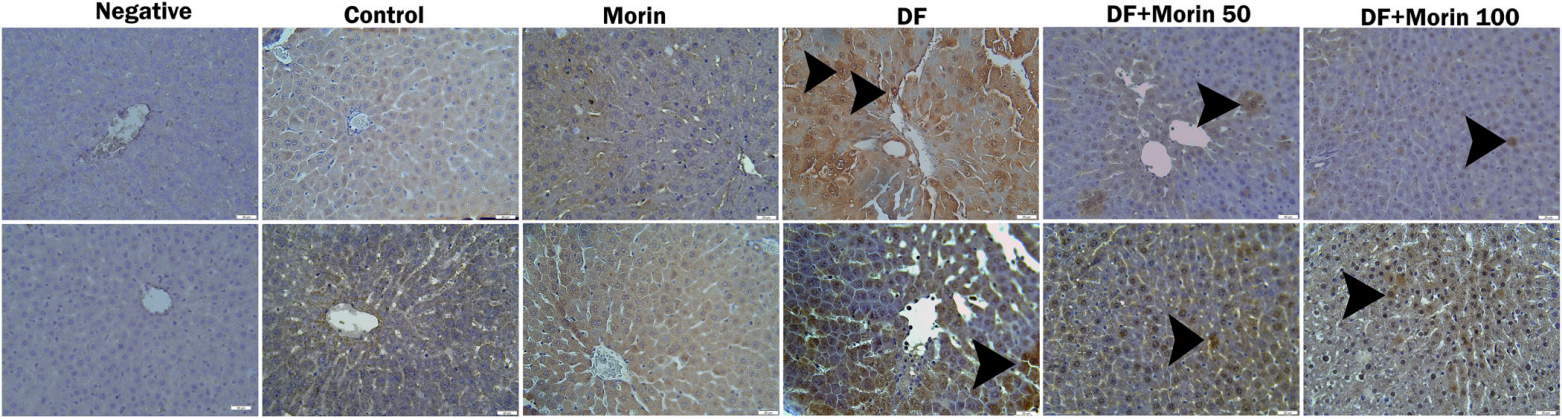
Protective effects of Morin and DF treatments on 8‐OHdG and Caspase‐3 immunopositivity in liver tissue. Liver tissue of DF (Diclofenac), Morin + DF 50 (Diclofenac + Morin 50) and Morin + DF 100 (Diclofenac + Morin 100) treated rats showed intense immunopositive cells (arrowhead) for 8‐OHdG and caspase‐3. Top row: 8‐OHdG, bottom row caspase‐3.

Caspase‐3 and 8‐OHdG immunohistochemistry results in liver tissue treated with DF and morin are given in Figure 7 and Table 3. According to liver tissue immunohistochemistry staining results, caspase‐3 and 8‐OHdG reactions were not observed in most sections in the control and morin groups (Figure 7). The caspase‐3 and 8‐OHdG immune reaction was observed to be severe in the DF‐administered group. There was a significant decrease in caspase‐3 and 8‐OHdG immune reactions in the DF+Morin 50 and DF+Morin 100 groups compared to the DF group (*p* < 0.05).

**Table 3:** Histopathological and immunohistochemical findings and their scores in liver tissue.

Parameters	Control	Morin	DF	DF + Morin 50	DF + Morin 100
Necrosis in hepatocytes	0.14 ± 0.37	0.14 ± 0.37^#^	2.42 ± 0.53*	1.14 ± 0.37*^#^	0.85 ± 0.37*^#^
Hyperemia, bleeding in interstitial vessels	0.28 ± 0.48	0.14 ± 0.37^#^	2.85 ± 0.37*	1.28 ± 0.48*^#^	1.14 ± 0.37*^#^
Inflammatory cell infiltration	0.14 ± 0.37	0.14 ± 0.37^#^	2.42 ± 0.53*	0.71 ± 0.48*^#^	0.42 ± 0.53*^#^
8‐OHdG expression	0.14 ± 0.37	0^#^	2.14 ± 0.37*	1.28 ± 0.48*^#^^⁎^	0.42 ± 0.53*^#^
Caspase‐3 expression	0.28 ± 0.48	0.14 ± 0.37^#^	2.42 ± 0.53*	1.42 ± 0.53*^#^	1.14 ± 0.37*^#^

*Note:* Control vs others: *p < 0.05, DF vs others: #*p* < 0.05, DF + Morin 50 vs DF + Morin 100: ^⁎^ *p* < 0.05.

### 3.9 Histopathological Results

**Figure 8:**
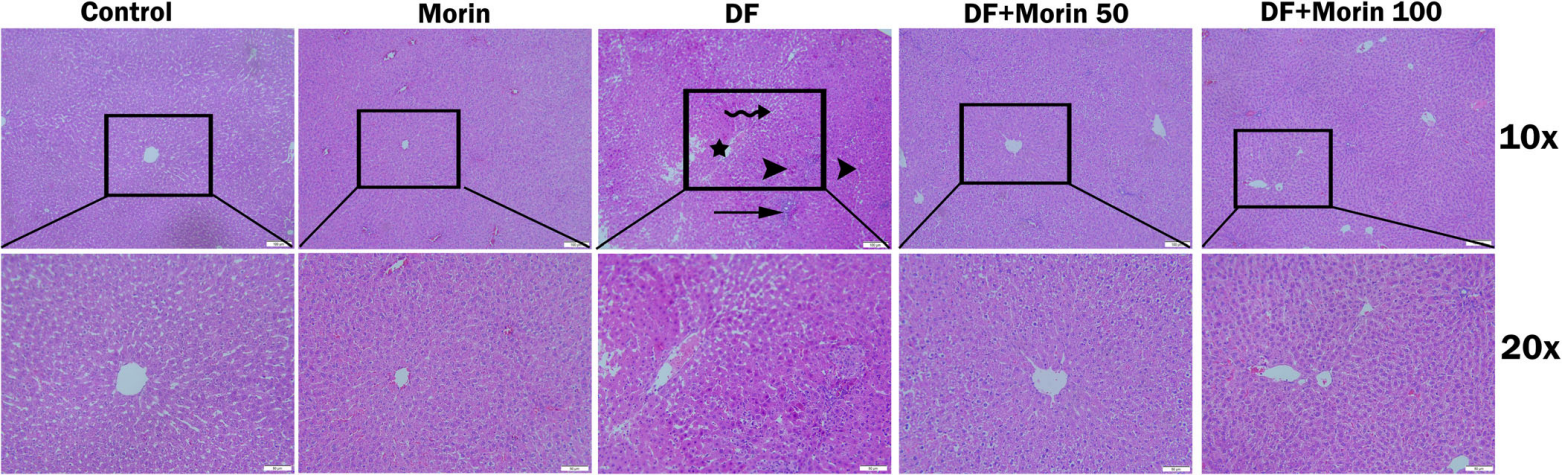
Photomicrographs of liver tissue histological changes (H&E staining). Control group, Morin group, DF (Diclofenac) group: arrowhead: necrosis of hepatocyte cells, curved arrow: sinusoidal dilatation, star: hyperemia and congestion in vessels, arrow: inflammatory cell infiltration, DF + Morin 50 (Diclofenac + Morin 50) group, DF + Morin 100 (Diclofenac + Morin 100) group.

Histological changes on liver tissues after DF and morin administration are shown in Figure 8 and Table 3. No pathological findings were found in the liver tissues in the control and morin groups. Hyperemia was noted in the liver tissue vessels and sinusoids of rats treated with DF. Additionally, inflammatory cell infiltration, sinusoidal dilatation and necrotic hepatocytes were observed. In the DF + Morin 50 and DF + Morin100 groups, hyperemia in the vessels was noted in rare areas. On the other hand, hepatocytes and lobules arranged in cords were found to be close to control. According to histopathological scoring results, rats in the DF group showed higher liver damage scores compared to the control group (*p* < 0.05). After DF application, the damage score decreased significantly in the treatment groups where Morin was applied compared to the DF group (*p* < 0.05). There was no significant difference between the two doses of DF treatment, although the higher dose appeared lower in the table.

## 4 Discussion

Diclofenac is a COX‐2 inhibitor with analgesic, anti‐inflammatory and antipyretic effects [7]. Although safe at therapeutic doses, a single overdose or unconscious use of consecutive high doses causes acute toxicity to the liver. This may cause unwanted side effects and death in a short time.

The production of reactive oxygen species and reactive metabolites and the resulting mitochondrial dysfunction are the main reasons for diclofenac toxicity. Reactive metabolites produced in excess are likely generated via the cytochrome P‐450 (CYP) enzyme, which in this case catalyzes the oxidation of diclofenac, reactive metabolites (4’ hydroxyl 3 diclofenac, 5’ hydroxyl 4 diclofenac and 5’ hydroxyl 6 diclofenac), to reactive benzoquinone imines, which are detoxified [26]. Therefore, the use of natural agents with antioxidant effects may protect against liver damage. In light of this information, the curative effects of morin on DF‐induced hepatotoxicity were evaluated in the current study.

AST, ALT and ALP, which are the first markers used to detect liver damage in clinical and research centres, were significantly increased in the DF‐treated group compared to the control group. These enzymes, which are found at normal levels in the liver, are released into the circulation to an increased extent due to necrotic changes and membrane damage in the hepatocyte [27]. In this study, administration of morin at different doses for therapeutic purposes decreased AST, ALT and ALP activity, which may be related to the protective effect of morin by protecting hepatocyte membrane integrity and decreasing AST, ALT and ALP leakage into circulation. Previous studies have emphasized that morin administration has a therapeutic effect by reducing AST, ALT and ALP activities, which indicate liver function [28, 29].

Drug‐induced liver injury caused by diclofenac can be explained by oxidative stress triggered due to excessive production of reactive oxygen species (ROS) and insufficiency of antioxidants [3]. MDA, an important indicator of lipid peroxidation (LPO), has been reported to be induced by diclofenac in previous studies [5, 30]. In the current study, MDA level in rats increased significantly as a result of DF administration, while treatment with morin caused a decrease in MDA content. As a result, the protective effect of morin on drug‐induced lipid peroxidation can be attributed to its ability to neutralize free radicals.

The oxidative stress process is triggered when the ROS level exceeds the antioxidant capacity and damages DNA, lipids and proteins [31]. Antioxidants exist in two forms, enzymatic and Nonenzymatic, and are necessary to scavenge ROS. Nonenzymatic reduced GSH is an endogenous antioxidant that maintains redox balance. It is also a substrate for GPx, which reduces lipid hydroperoxide radicals to hydroxy‐fatty acids [5, 32]. Previous experimental studies on different tissues have determined that DF can induce oxidative stress by reducing the intracellular level of GSH [5, 8]. In this study, it was revealed that DF depleted the GSH level in the liver, while morin caused a significant improvement in GSH content. This may be due to the ability of DF to increase GSH levels or the protective effect of the drug against oxidative stress. To understand the effect of morin on GSH metabolism in the current study, its effect on GPx, an enzyme that deactivates peroxides by converting GSH to oxidized glutathione (GSSG), was investigated. According to the data, it was revealed that DF probably induces oxidative stress by causing a decrease in GPx activity, while morin activates GPx.

Antioxidants with enzyme structure are generally expressed in the cytoplasm, mitochondria and peroxisome [33, 34, 35]. SOD and CAT, which are vital enzymes of the antioxidant system, have the task of creating H_2_O_2_ from superoxide radicals (O_2_^−^) and converting H_2_O_2_ into H_2_O and oxygen molecules, respectively [11]. Various studies have shown that DF reduces antioxidant activities in the liver. It has also been reported that DF forms reactive metabolites such as 5‐hydroxyl and N,5‐dihydroxyl, which may cause oxidative damage in the liver [5, 26]. Additionally, In Vivo studies have shown that morin provides protection by increasing SOD and CAT activities [12, 36, 37]. In the current study, DF administration caused a decrease in SOD and CAT activities, which may be an indicator of drug‐induced oxidative damage. However, it was revealed that morin administration caused a significant increase in SOD and CAT activities. It was concluded that the hydroxyl group in the morin structure probably fills GSH stores by scavenging free radicals, increases antioxidant enzymes by increasing the expression of SOD, CAT, GPx and reduces MDA levels by reducing oxidative stress. In this case, it was determined that morin provides protection against oxidative stress caused by DF by having a modulatory effect and improves the tissue antioxidant status. Cells have phase II detoxifying enzymes along with antioxidant proteins to counter oxidative stress attacks [3, 38]. Nrf2, identified as the major protection mechanism that neutralizes oxidative stress, is a redox‐sensitive transcription factor. The mechanism of Nrf2 protection is by regulating oxidative and inflammatory responses through the induction of phase II deoxidizing enzymes such as HO‐1 and NADPH dehydrogenase quinone‐1 [39, 40, 41]. In a previous study conducted on mice, it was observed that mice were hypersensitive to oxidative stress as a result of knockout of Nrf2, and as a result, it was reported that it had a vital role in maintaining Nrf2‐mediated intracellular redox homeostasis [42]. In the current study, it has been shown that DF administration inhibits Nrf2/HO‐1/NQO1 expression and thus causes liver damage. This result was supported by studies showing that DF‐induced increased ROS production suppressed the expression of Nrf2 and HO‐1 in different tissues of rats [30, 43]. It has been reported that administration of morin and DF together increases Nrf2/HO‐1/NQO1 expression. It was concluded that morin modulates oxidative stress that causes DF‐induced liver toxicity by activating the Nrf2 pathway.

Selective COX‐2 inhibitors have been withdrawn from the market because they pose a great risk to vital organs. NSAID class COX‐2 inhibitors have previously been proven to be hepatotoxic [3, 4, 5]. In this study, the effect of diclofenac, which inhibits prostaglandin synthesis by blocking cyclooxygenase enzymes, on COX‐2 RNA expression was investigated. As expected, diclofenac administration was found to inhibit COX‐2 expression. Additionally, it was determined that morin treatment had an activating effect on COX‐2, reversing the effect of diclofenac. This effect may be attributed to NF‐κB inhibition since NF‐κB activation has been shown to be at the head of the pro‐inflammatory cytokine induction pathway [17]. Previous studies have found that the anti‐inflammatory effect of morin is associated with the inhibition of the NF‐κB signaling pathway and therefore modulating COX‐2 [17, 36].

Accumulating evidence has reported that diclofenac causes oxidative stress‐induced cell death [7, 8]. There are two different cell death mechanisms in cells: autophagy and apoptosis. While autophagy is generally a mechanism that supports cell survival, apoptosis is associated with programmed cell death. The interaction between these two pathways occurs through various regulatory proteins. Autophagy is basically an effective mechanism to control cellular homeostasis. Under stress, with the increase in damaged organelles and misfolded proteins in cells, autophagy is overstimulated, causing structural and functional disorders [44]. The three most common major proteins used to study autophagy are beclin 1, LC3A and LC3B. Beclin 1 is a scaffolding protein for autophogosome nucleation and formation of the PI3K complex. In addition, Beclin‐1 is a key protein that initiates autophagy and also modulates apoptosis by interacting with Bcl‐2 family members. Bcl‐2 inhibits autophagy when bound with Beclin‐1, while suppressing the proapoptotic Bax protein in the apoptosis pathway. This bidirectional regulation complicates the role of Beclin‐1 in both autophagy and apoptosis. Furthermore, caspase‐3, which is activated during apoptosis, cleaves Beclin‐1, stopping autophagy and directing the cell to apoptosis.

LC3A, one of the LC3 forms that is an important marker that contributes to the formation of autophogosomes, is converted to the LC3B form by conjugation with phosphatidylethanolamine [44, 45]. According to the results of the present study, DF exposure caused an increase in Beclin 1, LC3A and LC3B levels, while these effects were reversed due to morin administration. In conclusion, morin decreased the increased cell death induced by DF‐induced liver injury; thus, it was concluded that morin may have an antiotophagic effect.

Another biological process that is vital for the establishment and maintenance of homeostatic balance in cells is apoptosis [46, 47]. In general, proteins such as apoptotic factors, p53, caspases and cytochrome play a role in the regulation of apoptosis [48]. When the balance between apaptotic factors consisting of proapoptotic and antiapoptotic members is disrupted, they induce apoptosis by increasing the release of Cytochrome‐c and apoptosis inducing factor (AIF) [49, 50]. Cytochrome‐c released from mitochondrial pores forms a complex called apoptosome in the cytosol with the participation of Apaf‐1 and ATP. Apoptosis cuts and activates caspase 9. Caspase‐9 turns procaspase‐3 into active caspase‐3 and the process of apoptosis begins [47, 51, 52, 53]. It has been reported that diclofenac suppresses antiapoptotic factors and damages tissues by activating proapaptotic factors [30, 54, 55]. In the present study, it was found that diclofenac increased the mRNA expression of p53, caspase‐3, caspase 6, caspase 9, Apaf‐1, bax, Bcl2 and cytochrome c, caspase 3 protein levels in rat livers and decreased Bcl2 mRNA expression. Morin, on the other hand, has an antiapoptotic effect by activating the Bcl‐2 gene and suppressing Bax, Caspase‐3, caspase 6, caspase 9, apaf, cytochrome c and p53 gene expressions. Likewise, immunohistochemical study results showed that caspase‐3 levels were consistent with PCR studies. This can be explained by the suppression of apoptosis following the reduction of oxidative stres. These results suggest that morin promotes cell survival by activating autophagy while reducing cell death by suppressing the apoptosis process. Thus, morin shifts the balance between autophagy and apoptosis in favor of survival and can protect hepatocytes against diclofenac‐induced damage.

MAPK is an important signaling pathway that plays a role in many processes in cells, including development, cell division, hormone responses and stress responses. It has been reported that MAPK14, MAPK15 and JNK play an active role as osmoregulators in the process of cell division, protein secretion and apoptosis, especially in cases of stress [27, 56, 57, 58]. According to the current study findings, MAPK14, MAPK15 and JNK expression levels increased with DF exposure, and expression levels decreased as a result of treatment with morin. In previous studies, it has been emphasised that morin has a suppressive effect on MAPK14, MAPK15 and JNK [27, 29, 59].

The guanine base of 8‐OHdG, an important marker of oxidative DNA damage, is highly sensitive to free radicals. It is known that increased reactive oxygen species oxidize guanine and cause DNA breaks [60, 61, 62]. Previously, Yanaka et al. In their study on diclofenac, it was documented that 8‐OHdG increased [63]. In the current animal study, it was determined that the 8‐OHdG immune reaction increased with DF administration, and this reaction decreased with morin administration.

In the presented study, liver biochemical results were confirmed by histological findings. In their experimental study by Esmaeilzadeh et al., it was found that there were serious histological lesions in the livers of rats given diclofenac [11]. In this study, it was proven that DF administration disrupted the liver morphology in light microscopic findings, with inflammatory cell infiltration, necrotic changes and vacuolization. In the rat group treated with morin, it was determined that the liver histological structure was largely preserved.

## 5 Conclusion

In conclusion, this study demonstrated that morin showed protective effects against DF‐induced liver injury by suppressing oxidative stress, apoptosis and autophagy pathways in rat liver tissue. This supports the hypothesis that the combination of morin with DF may provide a therapeutic effect on liver toxicity. This study provides important In Vivo data on the interactions between morin and diclofenac; however, the lack of a reference hepatoprotective agent limits the comparative assessment of the protective effects of morin. In addition, the lack of In Vitro experiments prevents clarification of the cellular level mechanisms of the observed effects. In future studies, comparison with a standard reference agent, addition of In Vitro analyses in models such as hepatocytes, and analysis of signals with advanced methods will establish the efficacy of morin on a more solid basis.

## Author Contributions

**Nurhan Akaras:** research, methodology, investigation, histopathological examination. **Hasan Şimşek:** methodology, investigation, data curation, formal analysis. **Cihan Gür:** methodology, investigation, data curation, formal analysis. **Mustafa İleritürk:** methodology, investigation, data curation, formal analysis. **Sefa Küçükler:** methodology, investigation, data curation, formal analysis. **Fatih Mehmet Kandemir:** investigation, formal analysis, supervision. **Nurhan Akaras:** wrote the first draft of the article. All authors read and approved the final manuscript.

## Ethics Statement

Ethics committee approval was received for this study from Necmettin Erbakan University KONUDAM local ethics committee for animal experiments (No: 2022/056).

## Consent

The authors have nothing to report.

## Conflicts of Interest

The authors declare no conflicts of interest.

## Data Availability

The data that support the findings of this study are available from the corresponding author upon reasonable request.

## References

[1] N. Aljuhani , M. A. Elkablawy , H. M. Elbadawy , et al., “Protective Effects of Ajwa Date Extract Against Tissue Damage Induced by Acute Diclofenac Toxicity,” Journal of Taibah University Medical Sciences 14, no. 6 (2019): 553–559. 31908644 10.1016/j.jtumed.2019.10.002 PMC6940670

[2] F. M. Steckling , F. D. Lima , J. B. Farinha , et al., “Diclofenac Attenuates Inflammation Through TLR4 Pathway and Improves Exercise Performance After Exhaustive Swimming,” Scandinavian Journal of Medicine & Science in Sports 30, no. 2 (2020): 264–271. 31618484 10.1111/sms.13579

[3] R. Chaabani , M. Bejaoui , I. Ben Jeddou , et al., “Effect of the Non‐Steroidal Anti‐Inflammatory Drug Diclofenac on Ischemia‐Reperfusion Injury in Rat Liver: A Nitric Oxide‐Dependent Mechanism,” Inflammation 46, no. 4 (2023): 1221–1235. 36933163 10.1007/s10753-023-01802-9

[4] C. Tu , Y. Gao , D. Song , et al., “Screening for Susceptibility‐Related Biomarkers of Diclofenac‐Induced Liver Injury in Rats Using Metabolomics,” Frontiers in Pharmacology 12 (2021): 693928. 34630079 10.3389/fphar.2021.693928 PMC8494976

[5] G. S. Villa‐Jaimes , H. Moshage , F. J. Avelar‐González , et al., “Molecular and Antioxidant Characterization of *Opuntia robusta* Fruit Extract and Its Protective Effect Against Diclofenac‐Induced Acute Liver Injury in an In Vivo Rat Model,” Antioxidants 12, no. 1 (2023): 113. 36670975 10.3390/antiox12010113 PMC9855095

[6] E. Heidarian and A. Nouri , “Hepatoprotective Effects of Silymarin Against Diclofenac‐Induced Liver Toxicity in Male Rats Based on Biochemical Parameters and Histological Study,” Archives of Physiology and Biochemistry 127, no. 2 (2021): 112–118. 31165636 10.1080/13813455.2019.1620785

[7] F. A. Aguilar Mora , N. Musheshe , A. Oun , et al., “Elevated cAMP Protects Against Diclofenac‐Induced Toxicity in Primary Rat Hepatocytes: A Protective Effect Mediated by the Exchange Protein Directly Activated by cAMP/cAMP‐Regulated Guanine Nucleotide Exchange Factors,” Molecular Pharmacology 99, no. 4 (2021): 294–307. 33574047 10.1124/molpharm.120.000217 PMC11033960

[8] R. A. Hassan , W. G. Hozayen , H. T. Abo Sree , H. M. Al‐Muzafar , and K. A. Amin , “O.M. Naringin and Hesperidin Counteract Diclofenac‐Induced Hepatotoxicity in Male Wistar Rats via Their Antioxidant, Anti‐Inflammatory, and Antiapoptotic Activities,” Oxidative Medicine and Cellular Longevity 2021 (2021): 9990091. 34422219 10.1155/2021/9990091 PMC8376442

[9] K. Riane , M. Sifour , H. Ouled‐Haddar , C. Espinosa , M. A. Esteban , and M. Lahouel , “Effect of Probiotic Supplementation on Oxidative Stress Markers in Rats With Diclofenac‐Induced Hepatotoxicity,” Brazilian Journal of Microbiology 51, no. 4 (2020): 1615–1622. 32458261 10.1007/s42770-020-00302-4 PMC7688739

[10] P. N. Thai , L. Ren , W. Xu , et al., “Chronic Diclofenac Exposure Increases Mitochondrial Oxidative Stress, Inflammatory Mediators, and Cardiac Dysfunction,” Cardiovascular Drugs and Therapy 37, no. 1 (2023): 25–37. 34499283 10.1007/s10557-021-07253-4 PMC8904649

[11] M. Esmaeilzadeh , E. Heidarian , M. Shaghaghi , et al., “Gallic Acid Mitigates Diclofenac‐Induced Liver Toxicity by Modulating Oxidative Stress and Suppressing IL‐1β Gene Expression in Male Rats,” Pharmaceutical Biology 58, no. 1 (2020): 590–596. 32633182 10.1080/13880209.2020.1777169 PMC7470116

[12] M. Kuzu , S. Yıldırım , F. M. Kandemir , et al., “Protective Effect of Morin on Doxorubicin‐Induced Hepatorenal Toxicity in Rats,” Chemico‐Biological Interactions 308 (2019): 89–100. 31100273 10.1016/j.cbi.2019.05.017

[13] S. Çomaklı , F. M. Kandemir , S. Küçükler , and S. Özdemir , “Morin Mitigates Ifosfamide Induced Nephrotoxicity by Regulation of NF‐kappaB/p53 and Bcl‐2 Expression,” Biotechnic & Histochemistry 97, no. 6 (2022): 423–432. 35037524 10.1080/10520295.2021.2021449

[14] Y. Asadi‐Fard , M. Z. Soleimani , M. J. Khodayar , L. Khorsandi , M. Shirani , and A. Samimi , “Morin Improves Bisphenol‐A‐Induced Toxicity in the Rat Testicular Mitochondria and Sperms,” JBRA Assisted Reproduction 27, no. 2 (2023): 174–179. 36356172 10.5935/1518-0557.20220010 PMC10279436

[15] L. Sang , X. M. Wang , D. Y. Xu , L. X. Sang , Y. Han , and L. Y. Jiang , “Morin Enhances Hepatic Nrf2 Expression in a Liver Fibrosis Rat Model,” World Journal of Gastroenterology 23, no. 47 (2017): 8334–8344. 29307993 10.3748/wjg.v23.i47.8334 PMC5743504

[16] H. Çelik , S. Kucukler , S. Çomaklı , et al., “Morin Attenuates Ifosfamide‐Induced Neurotoxicity in Rats via Suppression of Oxidative Stress, Neuroinflammation and Neuronal Apoptosis,” Neurotoxicology 76 (2020): 126–137. 31722249 10.1016/j.neuro.2019.11.004

[17] C. Gur , F. M. Kandemir , E. Darendelioglu , et al., “Morin Protects Against Acrylamide‐Induced Neurotoxicity in Rats: An Investigation into Different Signal Pathways,” Environmental Science and Pollution Research 28, no. 36 (2021): 49808–49819. 33939091 10.1007/s11356-021-14049-4

[18] J. P. S and S. Evan Prince , “Diclofenac‐Induced Renal Toxicity in Female Wistar Albino Rats Is Protected by the Pre‐Treatment of Aqueous Leaves Extract of *Madhuca longifolia* Through Suppression of Inflammation, Oxidative Stress and Cytokine Formation,” Biomedicine & Pharmacotherapy 98 (2018): 45–51. 29245065 10.1016/j.biopha.2017.12.028

[19] Z. A. Placer , L. L. Cushman , and B. C. Johnson , “Estimation of Product of Lipid Peroxidation (Malonyl Dialdehyde) in Biochemical Systems,” Analytical Biochemistry 16, no. 2 (1966): 359–364. 6007581 10.1016/0003-2697(66)90167-9

[20] J. Sedlak and R. H. Lindsay , “Estimation of Total, Protein‐Bound, and Nonprotein Sulfhydryl Groups in Tissue With Ellman's Reagent,” Analytical Biochemistry 25, no. 1 (1968): 192–205. 4973948 10.1016/0003-2697(68)90092-4

[21] R. A. Lawrence and R. F. Burk , “Glutathione Peroxidase Activity in Selenium‐Deficient Rat Liver,” Biochemical and Biophysical Research Communications 71, no. 4 (1976): 952–958. 971321 10.1016/0006-291x(76)90747-6

[22] Y. Sun , L. W. Oberley , and Y. Li , “A Simple Method for Clinical Assay of Superoxide Dismutase,” Clinical Chemistry 34, no. 3 (1988): 497–500. 3349599

[23] H. Aebi , “Catalase In Vitro,” Methods in Enzymology 105 (1984): 121–126. 6727660 10.1016/s0076-6879(84)05016-3

[24] O. H. Lowry , N. J. Rosebrough , and A. L. Farr , “R.J. Protein Measurement With the Folin Phenol Reagent,” Journal of Biology Chemistry 193, no. 1 (1951): 265–275. 14907713

[25] K. J. Livak and T. D. Schmittgen , “Analysis of Relative Gene Expression Data Using Real‐Time Quantitative PCR and the 2(‐Delta Delta C(T)) Method,” Methods (San Diego, Calif.) 25, no. 4 (2001): 402–408. 11846609 10.1006/meth.2001.1262

[26] Q. K. Alabi , R. O. Akomolafe , O. S. Olukiran , et al., “The *Garcinia kola* Biflavonoid Kolaviron Attenuates Experimental Hepatotoxicity Induced by Diclofenac,” Pathophysiology 24, no. 4 (2017): 281–290. 28822616 10.1016/j.pathophys.2017.07.003

[27] H. E. Kızıl , C. Caglayan , E. Darendelioğlu , et al., “Morin Ameliorates Methotrexate‐Induced Hepatotoxicity via Targeting Nrf2/Ho‐1 and Bax/Bcl2/Caspase‐3 Signaling Pathways,” Molecular Biology Reports 50, no. 4 (2023): 3479–3488. 36781607 10.1007/s11033-023-08286-8

[28] S. Özdemir , S. Kucukler , S. Çomaklı , and F. M. Kandemir , “The Protective Effect of Morin Against Ifosfamide‐Induced Acute Liver Injury in Rats Associated With the Inhibition of DNA Damage and Apoptosis,” Drug and Chemical Toxicology 45, no. 3 (2022): 1308–1317. 32957801 10.1080/01480545.2020.1822390

[29] F. M. Kandemir , S. Yıldırım , S. Kucukler , C. Caglayan , E. Darendelioğlu , and M. B. Dortbudak , “Protective Effects of Morin Against Acrylamide‐Induced Hepatotoxicity and Nephrotoxicity: A Multi‐Biomarker Approach,” Food and Chemical Toxicology 138 (2020): 111190. 32068001 10.1016/j.fct.2020.111190

[30] B. Varışlı , C. Caglayan , F. M. Kandemir , et al., “Chrysin Mitigates Diclofenac‐Induced Hepatotoxicity by Modulating Oxidative Stress, Apoptosis, Autophagy and Endoplasmic Reticulum Stress in Rats,” Molecular Biology Reports 50, no. 1 (2023): 433–442. 36344803 10.1007/s11033-022-07928-7

[31] E. H. Aksu , F. M. Kandemir , S. Yıldırım , et al., “Palliative Effect of Curcumin on Doxorubicin‐Induced Testicular Damage in Male Rats,” Journal of Biochemical and Molecular Toxicology 33, no. 10 (2019): e22384. 31468665 10.1002/jbt.22384

[32] N. Akaras , M. Ileriturk , C. Gur , S. Kucukler , M. Oz , and F. M. Kandemir , “The Protective Effects of Chrysin on Cadmium‐Induced Pulmonary Toxicity; a Multi‐Biomarker Approach,” Environmental Science and Pollution Research 30, no. 38 (2023): 89479–89494. 37453011 10.1007/s11356-023-28747-8

[33] A. Nouri , F. Izak‐Shirian , V. Fanaei , et al., “Carvacrol Exerts Nephroprotective Effect in Rat Model of Diclofenac‐Induced Renal Injury Through Regulation of Oxidative Stress and Suppression of Inflammatory Response,” Heliyon 7, no. 11 (2021): e08358. 34816045 10.1016/j.heliyon.2021.e08358 PMC8591494

[34] N. Akaras , O. O. Abuc , K. Koc , et al., “1 → 3)‐β‐d‐glucan Enhances the Toxicity Induced by Bortezomib in Rat Testis,” Journal of Food Biochemistry 44, no. 3 (2020): e13155. 31960484 10.1111/jfbc.13155

[35] O. Keleş , S. Can , G. Cigsar , et al., “Hepatoprotective Effects of B‐1, 3‐(D)‐glucan on Bortezomib‐induced Liver Damage in Rats,” Kafkas Üniversitesi Veteriner Fakültesi Dergisi 20, no. 6 (2014): 929–938.

[36] S. Kucukler , C. Caglayan , E. Darendelioğlu , and F. M. Kandemir , “Morin Attenuates Acrylamide‐Induced Testicular Toxicity in Rats by Regulating the NF‐κB, Bax/Bcl‐2 and PI3K/Akt/mTOR Signaling Pathways,” Life Sciences 261 (2020): 118301. 32827546 10.1016/j.lfs.2020.118301

[37] F. Cakmak , S. Kucukler , C. Gur , S. Comakli , M. Ileriturk , and F. M. Kandemir , “Morin Provides Therapeutic Effect by Attenuating Oxidative Stress, Inflammation, Endoplasmic Reticulum Stress, Autophagy, Apoptosis, and Oxidative DNA Damage in Testicular Toxicity Caused by Ifosfamide in Rats,” Iranian Journal of Basic Medical Sciences 26, no. 10 (2023): 1227–1236. 37736509 10.22038/IJBMS.2023.71702.15580 PMC10510477

[38] E. H. Aksu , F. M. Kandemir , S. Küçükler , and A. Mahamadu , “Improvement in Colistin‐Induced Reproductive Damage, Apoptosis, and Autophagy in Testes via Reducing Oxidative Stress by Chrysin,” Journal of Biochemical and Molecular Toxicology 32, no. 11 (2018): e22201. 30273961 10.1002/jbt.22201

[39] S. A. Akarsu , C. Gür , M. Ileritürk , N. Akaras , S. Küçükler , and F. M. Kandemir , “Effect of Syringic Acid on Oxidative Stress, Autophagy, Apoptosis, Inflammation Pathways Against Testicular Damage Induced by Lead Acetate,” Journal of Trace Elements in Medicine and Biology 80 (2023): 127315. 37801787 10.1016/j.jtemb.2023.127315

[40] H. Şimşek , C. Gür , S. Küçükler , et al., “Carvacrol Reduces Mercuric Chloride‐Induced Testicular Toxicity by Regulating Oxidative Stress, Inflammation, Apoptosis, Autophagy, and Histopathological Changes,” Biological Trace Element Research 202, no. 10 (2024): 4605–4617. 38133725 10.1007/s12011-023-04022-2

[41] N. Akaras , C. Gür , C. Caglayan , and F. M. Kandemir , “Protective Effects of Naringin Against Oxaliplatin‐Induced Testicular Damage in Rats: Involvement of Oxidative Stress, Inflammation, Endoplasmic Reticulum Stress, Apoptosis, and Histopathology,” Iranian Journal of Basic Medical Sciences 27, no. 4 (2024): 466–474. 38419883 10.22038/IJBMS.2024.73824.16048 PMC10897554

[42] Z. A. Shah , R. C. Li , R. K. Thimmulappa , et al., “Role of Reactive Oxygen Species in Modulation of Nrf2 Following Ischemic Reperfusion Injury,” Neuroscience 147, no. 1 (2007): 53–59. 17507167 10.1016/j.neuroscience.2007.02.066 PMC1961622

[43] C. Boonyong , N. Vardhanabhuti , and S. Jianmongkol , “Modulation of Non‐Steroidal Anti‐Inflammatory Drug‐Induced, ER Stress‐Mediated Apoptosis in Caco‐2 Cells by Different Polyphenolic Antioxidants: A Mechanistic Study,” Journal of Pharmacy and Pharmacology 72, no. 11 (2020): 1574–1584. 32716561 10.1111/jphp.13343

[44] N. A. Kankılıç , H. Şimşek , N. Akaras , et al., “Protective Effects of Naringin on Colistin‐Induced Damage in Rat Testicular Tissue: Modulating the Levels of Nrf‐2/HO‐1, AKT‐2/FOXO1A, Bax/Bcl2/Caspase‐3, and Beclin‐1/LC3A/LC3B Signaling Pathways,” Journal of Biochemical and Molecular Toxicology 38, no. 2 (2024): e23643. 38348713 10.1002/jbt.23643

[45] H. Şimşek , N. Akaras , C. Gür , S. Küçükler , and F. M. Kandemir , “Beneficial Effects of Chrysin on Cadmium‐Induced Nephrotoxicity in Rats: Modulating the Levels of Nrf2/Ho‐1, RAGE/NLRP3, and Caspase‐3/Bax/Bcl‐2 Signaling Pathways,” Gene 875 (2023): 147502. 37224935 10.1016/j.gene.2023.147502

[46] S. Yilmaz , C. Gur , S. Kucukler , N. Akaras , and F. M. Kandemir , “Zingerone Attenuates Sciatic Nerve Damage Caused by Sodium Arsenite by Inhibiting NF‐κB, caspase‐3, and ATF‐6/CHOP Pathways and Activating the Akt2/FOXO1 Pathway,” Iranian Journal of Basic Medical Sciences 27, no. 4 (2024): 485–491. 38419893 10.22038/IJBMS.2023.74088.16094 PMC10897565

[47] N. Akaras , S. Kucukler , C. Gur , M. Ileriturk , and F. M. Kandemir , “Sinapic Acid Protects Against Lead Acetate‐Induced Lung Toxicity by Reducing Oxidative Stress, Apoptosis, Inflammation, and Endoplasmic Reticulum Stress Damage,” Environmental Toxicology 39, no. 7 (2024): 3820–3832. 38530053 10.1002/tox.24255

[48] M. Ileriturk , D. Ileriturk , O. Kandemir , et al., “Naringin Attenuates Oxaliplatin‐Induced Nephrotoxicity and Hepatotoxicity: A Molecular, Biochemical, and Histopathological Approach in a Rat Model,” Journal of Biochemical and Molecular Toxicology 38, no. 1 (2024): e23604. 38037725 10.1002/jbt.23604

[49] F. N. Ekinci Akdemir , S. Yildirim , F. M. Kandemir , et al., “The Effects of Casticin and Myricetin on Liver Damage Induced by Methotrexate in Rats,” Iranian Journal of Basic Medical Sciences 21, no. 12 (2018): 1281–1288. 30627373 10.22038/ijbms.2018.29922.7217 PMC6312684

[50] K. Yesildag , C. Gur , M. Ileriturk , and F. M. Kandemir , “Evaluation of Oxidative Stress, Inflammation, Apoptosis, Oxidative DNA Damage and Metalloproteinases in the Lungs of Rats Treated With Cadmium and Carvacrol,” Molecular Biology Reports 49, no. 2 (2022): 1201–1211. 34792728 10.1007/s11033-021-06948-z

[51] S. A. Akarsu , M. İleritürk , S. Küçükler , N. Akaras , C. Gür , and F. M. Kandemir , “Ameliorative Effects of Sinapic Acid Against Vancomycin‐Induced Testicular Oxidative Damage, Apoptosis, Inflammation, Testicular Histopathologic Disorders and Decreased Epididymal Sperm Quality,” Reproductive Toxicology 129 (2024): 108666. 39059777 10.1016/j.reprotox.2024.108666

[52] K. S. Hashem , A. Z. Abdelazem , M. A. Mohammed , et al., “RETRACTED ARTICLE: Thymoquinone Alleviates Mitochondrial Viability and Apoptosis in Diclofenac‐Induced Acute Kidney Injury (AKI) Via Regulating Mfn2 and miR‐34a mRNA Expressions,” Environmental Science and Pollution Research 28, no. 8 (2021): 10100–10113. 33165700 10.1007/s11356-020-11313-x

[53] A. Tanyeli , E. Eraslan , M. C. Güler , N. Kurt , and N. Akaras , “Gossypin Protects Against Renal Ischemia‐Reperfusion Injury in Rats,” Kafkas Universitesi Veteriner Fakultesi Dergisi 26, no. 1 (2020): 89–96.

[54] S. N. Özbolat and A. Ayna , “Chrysin Suppresses HT‐29 Cell Death Induced by Diclofenac Through Apoptosis and Oxidative Damage,” Nutrition and Cancer 73, no. 8 (2021): 1419–1428. 32757685 10.1080/01635581.2020.1801775

[55] A. Dogra , D. Kour , A. Gour , et al., “Ameliorating Effect of Rutin Against Diclofenac‐Induced Cardiac Injury in Rats With Underlying Function of FABP3, MYL3, and ANP,” Drug and Chemical Toxicology 46, no. 3 (2023): 597–608. 35509154 10.1080/01480545.2022.2069804

[56] E. D. Arisan , Z. Ergül , G. Bozdağ , et al., “Diclofenac Induced Apoptosis via Altering PI3K/Akt/MAPK Signaling Axis in HCT 116 More Efficiently Compared to SW480 Colon Cancer Cells,” Molecular Biology Reports 45, no. 6 (2018): 2175–2184. 30406888 10.1007/s11033-018-4378-2

[57] C. Caglayan , F. M. Kandemir , A. Ayna , C. Gür , S. Küçükler , and E. Darendelioğlu , “Neuroprotective Effects of 18β‐glycyrrhetinic Acid Against Bisphenol A‐Induced Neurotoxicity in Rats: Involvement of Neuronal Apoptosis, Endoplasmic Reticulum Stress and JAK1/STAT1 Signaling Pathway,” Metabolic Brain Disease 37, no. 6 (2022): 1931–1940. 35699857 10.1007/s11011-022-01027-z

[58] S. Qayyum , A. Jabeen , S. Ashraf , et al., “Oxadiazole Derivatives of Diclofenac as an Anti‐Proliferative Agent for B‐Cell Non‐Hodgkin Lymphoma: An In Vitro and In Silico Studies,” Medicinal Chemistry 20, no. 4 (2024): 443–451. 38279758 10.2174/0115734064290905231228110023

[59] A. R. Maiuri , A. B. Breier , J. D. Turkus , P. E. Ganey , and R. A. Roth , “Calcium Contributes to the Cytotoxic Interaction Between Diclofenac and Cytokines,” Toxicological Sciences 149, no. 2 (2016): 372–384. 26609140 10.1093/toxsci/kfv249 PMC4900219

[60] M. Fuchs , C. Viel , A. Lehto , H. Lau , and J. Klein , “Oxidative Stress in Rat Brain During Experimental Status Epilepticus: Effect of Antioxidants,” Frontiers in Pharmacology 14 (2023): 1233184. 37767398 10.3389/fphar.2023.1233184 PMC10520702

[61] V. Yadav , A. Krishnan , S. Zahiruddin , S. Ahmad , and D. Vohora , “Amelioration of Cyclophosphamide‐Induced DNA Damage, Oxidative Stress, and Hepato‐ and Neurotoxicity by *Piper longum* Extract in Rats: The Role of γH2AX and 8‐OHdG,” Frontiers in Pharmacology 14 (2023): 1147823. 36969834 10.3389/fphar.2023.1147823 PMC10036401

[62] S. A. Akarsu , C. Gür , S. Küçükler , N. Akaras , M. İleritürk , and F. M. Kandemir , “Protective Effects of Syringic Acid Against Oxidative Damage, Apoptosis, Autophagy, Inflammation, Testicular Histopathologic Disorders, and Impaired Sperm Quality in the Testicular Tissue of Rats Induced by Mercuric Chloride,” Environmental Toxicology 39, no. 10 (2024): 4803–4814. 39096083 10.1002/tox.24395

[63] A. Yanaka , S. Zhang , D. Sato , et al., “Geranylgeranylacetone Protects the Human Gastric Mucosa From Diclofenac‐Induced Injury via Induction of Heat Shock Protein 70,” Digestion 75, no. 2–3 (2007): 148–155. 17684364 10.1159/000106756

